# Impact of Lipid Genetic Risk Score and Saturated Fatty Acid Intake on Central Obesity in an Asian Indian Population

**DOI:** 10.3390/nu14132713

**Published:** 2022-06-29

**Authors:** Ramatu Wuni, Evelyn Adela Nathania, Ashok K. Ayyappa, Nagarajan Lakshmipriya, Kandaswamy Ramya, Rajagopal Gayathri, Gunasekaran Geetha, Ranjit Mohan Anjana, Gunter G. C. Kuhnle, Venkatesan Radha, Viswanathan Mohan, Vasudevan Sudha, Karani Santhanakrishnan Vimaleswaran

**Affiliations:** 1 Hugh Sinclair Unit of Human Nutrition, Department of Food and Nutritional Sciences, University of Reading, Reading RG6 6DZ, UK; r.wuni@pgr.reading.ac.uk (R.W.); g.g.kuhnle@reading.ac.uk (G.G.C.K.); 2 Indonesia International Institute for Life Sciences, JI. Pulomas Barat Kav. 88, Jakarta Timur 13210, Indonesia; evelyn.nathania@student.i3l.ac.id; 3 Department of Molecular Genetics, Madras Diabetes Research Foundation, ICMR Centre for Advanced Research on Diabetes, Chennai 603103, India; ashokayyappa@gmail.com (A.K.A.); vkaranis2010@gmail.com (K.R.); dranjana@drmohans.com (R.M.A.); radharv@yahoo.co.in (V.R.); drmohans@diabetes.ind.in (V.M.); 4 Department of Food, Nutrition and Dietetics Research, Madras Diabetes Research Foundation, Chennai 600086, India; lakshmipriyasiva07@gmail.com (N.L.); gayathri@mdrf.in (R.G.); geetha@mdrf.in (G.G.); s2r_7@mdrf.in (V.S.); 5 Dr. Mohan’s Diabetes Specialties Centre, IDF Centre of Excellence in Diabetes Care, Gopalapuram, Chennai 600086, India; 6 The Institute for Food, Nutrition, and Health (IFNH), University of Reading, Reading RG6 6AP, UK

**Keywords:** genetic risk score, Asian Indians, lipids, central obesity, fat intake, gene-diet interaction, saturated fatty acid

## Abstract

Abnormalities in lipid metabolism have been linked to the development of obesity. We used a nutrigenetic approach to establish a link between lipids and obesity in Asian Indians, who are known to have a high prevalence of central obesity and dyslipidaemia. A sample of 497 Asian Indian individuals (260 with type 2 diabetes and 237 with normal glucose tolerance) (mean age: 44 ± 10 years) were randomly chosen from the Chennai Urban Rural Epidemiological Study (CURES). Dietary intake was assessed using a previously validated questionnaire. A genetic risk score (GRS) was constructed based on cholesteryl ester transfer protein (*CETP*) and lipoprotein lipase (*LPL*) genetic variants. There was a significant interaction between GRS and saturated fatty acid (SFA) intake on waist circumference (WC) (P_interaction_ = 0.006). Individuals with a low SFA intake (≤23.2 g/day), despite carrying ≥2 risk alleles, had a smaller WC compared to individuals carrying <2 risk alleles (Beta = −0.01 cm; *p* = 0.03). For those individuals carrying ≥2 risk alleles, a high SFA intake (>23.2 g/day) was significantly associated with a larger WC than a low SFA intake (≤23.2 g/day) (Beta = 0.02 cm, *p* = 0.02). There were no significant interactions between GRS and other dietary factors on any of the measured outcomes. We conclude that a diet low in SFA might help reduce the genetic risk of central obesity confirmed by *CETP* and *LPL* genetic variants. Conversely, a high SFA diet increases the genetic risk of central obesity in Asian Indians.

## 1. Introduction

Asian Indians are more prone to developing type 2 diabetes (T2D) and cardiovascular diseases (CVDs) at a lower body mass index (BMI) than Caucasians, due to the ‘Asian Indian phenotype’, which is characterised by central obesity, dyslipidaemia, and increased levels of total fat, visceral fat, insulin resistance and faster decline in beta cell function [1,2,3]. The location of body fat is thought to be more important in predicting adverse cardiovascular events [4,5,6]. Central obesity has been linked to several conditions, including insulin resistance and increased mortality from CVDs [7,8,9,10], necessitating studies to fully understand the underlying mechanisms for the development of central obesity in Asian Indians.

Abnormalities in lipid metabolism have been linked to the development of obesity, and lipoprotein lipase (LPL), a key enzyme in lipid metabolism, contributes to the development of obesity through its role in the partitioning of lipids to different tissues [10,11,12]. Cholesteryl ester transfer protein (CETP), mainly expressed in adipose tissue, is also a major enzyme in lipid metabolism, which mediates the transport of cholesteryl esters and triglycerides (TG) between high-density lipoprotein cholesterol (HDL) and apolipoprotein B (ApoB)–containing lipoproteins such as very-low-density lipoprotein (VLDL) [13]. Increased CETP activity results in lower HDL concentration, which is associated with higher risk of CVDs [14]. Consumption of a high saturated fatty acid (SFA) diet has also been shown to contribute to obesity by decreasing cholesterol efflux due to reduced expression of peroxisome proliferator-activated receptors involved in lipid metabolism [15,16,17]. Genome-wide association (GWA) and candidate gene studies have demonstrated that lipid levels are influenced by single nucleotide polymorphisms (SNPs) in lipid-pathway genes [18,19,20,21,22,23]. SNPs of the *CETP* gene have been associated with HDL concentrations [21,24,25,26,27,28], while SNPs of the *LPL* gene have been associated with both HDL and TG levels [21,29,30,31]. A recent review of GWA studies of lipids [32] showed that *CETP* SNPs had the highest number of associations with lipids, followed by *LPL* SNPs. *CETP* and *LPL* SNPs have also been associated with obesity-related traits [33,34].

Several studies have shown significant interactions between genetic variants and lifestyle factors regarding the association between lipid profile and obesity-related traits [1,19,33,35,36], but the findings have been inconsistent. Moreover, it has been shown that the effect size of individual SNPs is modest and less likely to accurately predict the risk of complex diseases, and a more effective approach involves combining several risk alleles to generate a genetic risk score (GRS) [35,37]. Nonetheless, studies investigating interactions between GRS and dietary factors on lipid and obesity-related traits have not been adequately performed in Asian Indians. Hence, the aim of this study was to examine the effect of a GRS and its interaction with dietary factors on lipid and obesity-related traits in Asian Indian adults with and without T2D.

## 2. Methods

### 2.1. Study Participants

**Figure 1:**
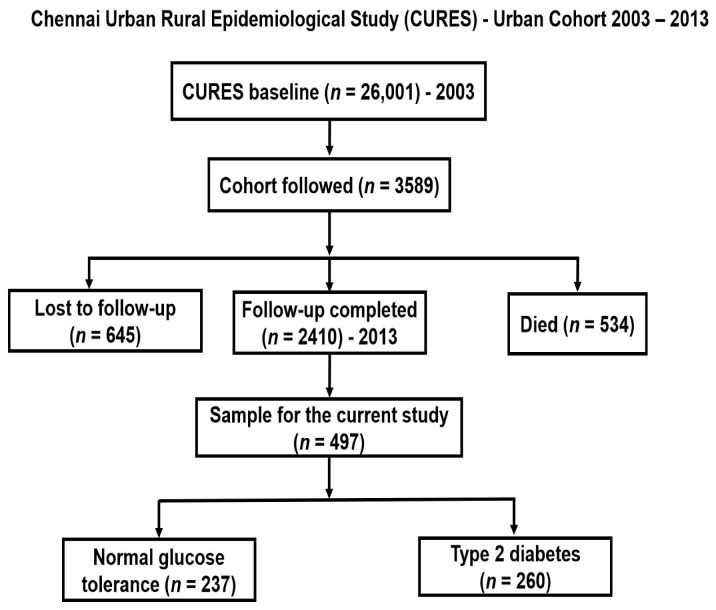
A flow chart showing the selection of participants from the CURES.

A sample of 497 individuals (260 with T2D and 237 with normal glucose tolerance (NGT)) were randomly chosen from an epidemiological study called the Chennai Urban Rural Epidemiological Study (CURES), details of which have been given in previous publications [1,19,33,35,38,39,40,41,42,43]. Briefly, a total of 26,001 adults residing in the urban part of Chennai in Southern India were recruited by systematic random sampling between 2001 to 2003, and those who reported having T2D (1529 individuals) were tested to confirm their diagnosis [1,40]. The follow-up study was conducted between 2012 to 2013 and consisted of 2410 participants (Figure 1). The sample for the current study was selected from the follow-up cohort. Participants were excluded if they were taking lipid-lowering medication such as statins and fibrates. Ethical approval was granted by the Madras Diabetes Research Foundation Institutional Ethics Committee and written informed consent was obtained from study participants [1].

### 2.2. Anthropometric and Biochemical Measurements

Anthropometric measurements including height, weight, waist circumference (WC), hip circumference, and waist–hip ratio (WHR) were obtained using standardized techniques. BMI was calculated as weight in kilograms (kg) divided by the square of the height in meters (m). Individuals with BMI < 25 kg/m^2^ were classified as non-obese and those with BMI ≥ 25 kg/m^2^ were classified as obese, in accordance with the World Health Organisation Asia Pacific Guidelines [44]. Biochemical analyses were conducted using Hitachi-912 Auto Analyzer (Hitachi, Mannheim, Germany) with kits supplied by Roche Diagnostics (Mannheim, Germany). Serum total cholesterol was measured by cholesterol oxidase-phenol-4-amino-antipyrene peroxidase method and HDL by direct method-polyethylene glycol-pretreated enzymes. Serum TG was measured by glycerol phosphatase oxidase-phenol-4-amino-antipyrene peroxidase method, and low-density lipoprotein cholesterol (LDL) was calculated using the Friedewald formula [45]. Serum insulin concentration was estimated using an enzyme-linked immunosorbent assay (Dako, Glostrup, Denmark), fasting plasma glucose (FPG) by glucose oxidase-peroxidase method, and glycated haemoglobin (HbA1c) by high-performance liquid chromatography using a Variant™ machine (Bio-Rad, Hercules, CA, USA).

### 2.3. Dietary Assessment

Dietary intake was assessed by an interviewer using a previously validated semi-quantitative food frequency questionnaire (FFQ) containing 222 items [46]. Participants were asked to estimate how much and how often they consumed various food items in the FFQ (number of times per day, week, month, year or never). The FFQ was designed to estimate the usual dietary intake of participants on a meal-by-meal basis. Open-ended questions were used to enable participants to estimate the frequency of their usual dietary intake. To help in estimating portion sizes, participants were shown common household measures such as spoons and cups and pictures of different sizes of fruits. The data were analysed using the Nutritional Epidemiology (‘EpiNu’) software to estimate average daily intake of macronutrients and total energy. Consumption of SFA, polyunsaturated fatty acid (PUFA), monounsaturated fatty acid (MUFA) and other macronutrients was estimated from the FFQ using the ‘EpiNu’ software which contains information on the nutritional content of commonly consumed food in the Chennai area. The ‘EpiNu’ software was developed for the local population using recipes from various sources including home-made and fast-food. Details of the development of the FFQ and the ‘EpiNu’ software are published elsewhere [46].

### 2.4. SNP Selection and Genotyping

Five SNPs (*CETP* SNP: rs4783961; and *LPL* SNPs: rs327, rs3200218, rs1800590 and rs268) were selected for this study based on their association with lipid-related traits in different ethnic groups, including Asian Indians [21,22,23,33,47,48,49,50]. Two SNPs (rs268 and rs1800590) had a minor allele frequency < 5% (Supplementary Table S1), and hence, they were excluded. The remaining three SNPs (rs327, rs3200218 and rs4783961) were included in the current analysis. The genotyping methodology has been previously published [19]. Briefly, the DNA was extracted from whole blood using the phenol–chloroform method, and the SNPs were genotyped by the polymerase chain reaction-restriction fragment length polymorphism method.

### 2.5. Construction of GRS

An additive model was used to construct an unweighted GRS by adding the number of risk alleles across the three SNPs (rs327, rs3200218 and rs4783961) for each participant. The risk alleles were defined as alleles previously reported to be associated with dyslipidaemia or obesity-related traits. The risk alleles were not weighed due to limited available information on effect sizes of the SNPs for the Asian Indian population. Moreover, it has been demonstrated that assigning weights to risk alleles only has minimal effect [37], and hence, we used an unweighted GRS. The 3-SNP GRS ranged from 0 to 5, and based on the median GRS (2 risk alleles), participants were placed into two groups: low-risk group (for individuals with a GRS < 2 risk alleles) and high-risk group (for individuals with a GRS ≥ 2 risk alleles).

### 2.6. Statistical Analysis

Statistical analysis was performed using Statistical Package for the Social Sciences (SPSS) software (version 28; SPSS Inc., Chicago, IL, USA). Normality test was performed by Shapiro–Wilk test, and all biochemical and anthropometric variables were log-transformed before the analysis. Results of descriptive statistics for continuous variables are presented as means and standard deviation (SD) and categorical variables as percentages [1]. Allele frequencies were determined by gene counting and a goodness-of-fit Chi-square test was performed to examine if the observed genotype counts were in Hardy-Weinberg equilibrium (HWE) (Supplementary Table S1). The three SNPs were all in HWE (*p* > 0.05), and the alleles had a frequency greater than 5%. An independent sample t test was used to compare the means of the quantitative variables between individuals with low GRS (<2 risk alleles) and those with high GRS (≥2 risk alleles). A Chi-square test was performed to compare categorical variables such as smoking status between individuals in the low (GRS < 2 risk alleles) and high-risk (GRS ≥ 2 risk alleles) groups.

Linear and logistic regression analyses were used to examine the association of the 3-SNP GRS with continuous and categorical outcomes, with adjustment for age, sex, BMI, T2D, duration of diabetes, anti-diabetic medication, smoking status, and alcohol intake wherever appropriate. Interactions between GRS and dietary factors were analysed by adding the interaction term in the regression models. For GRS–diet interactions, total energy was adjusted for, in addition to the other covariates. The dietary factors investigated in this study were consumption of fat, carbohydrate, protein, and dietary fibre. GRS–diet interactions reaching statistical significance (*p* < 0.05) were investigated further by stratifying individuals based on the quantity of dietary intake. A significant interaction of GRS with total fat was explored further to include subtypes of fats (SFA, PUFA and MUFA). A median intake of total fat, SFA, MUFA, and PUFA was used to classify individuals into two groups, ‘low’ (lower than median) and ‘high’ (higher than median) group, and association between GRS and the outcome was then analysed for each group.

## 3. Results

### 3.1. Characteristics of the Study Participants

The mean age of the study participants was 44 ± 10 (Table 1). At baseline, there were no significant differences in anthropometric traits (BMI, WC and WHR), lipid sub-fractions (HDL, LDL, TG, and total cholesterol), systolic blood pressure (SBP) and diastolic blood pressure (DBP), or glycaemic traits (FPG, fasting serum insulin, insulin resistance and HbA1c) between participants with low GRS (<2 risk alleles) and those with high GRS (≥2 risk alleles). Furthermore, consumption of macronutrients did not differ significantly between participants with low GRS (<2 risk alleles) and those with high GRS (≥2 risk alleles) as shown in Table 1. Smoking was higher among individuals with high GRS (≥2 risk alleles) compared to those with low GRS (<2 risk alleles) (*p* = 0.03). The baseline HDL concentration was significantly higher in women than in men (43.5 ± 1.3 vs. 38.7 ± 1.3 mg/dL; *p* = 2.3 × 10^−8^).

### 3.2. Association of GRS with Lipid and Obesity-Related Traits

There was no significant association between GRS and any of the outcomes measured (HDL, LDL, TG, total cholesterol, SBP, DBP, BMI, WC, WHR and obesity) after adjusting for the confounding factors, age, sex, BMI, T2D, duration of diabetes, anti-diabetic medication, smoking status, and alcohol intake where appropriate (Supplementary Tables S2 and S3).

### 3.3. Interaction of GRS with Dietary Factors on Lipid and Obesity Related Traits

**Figure 2:**
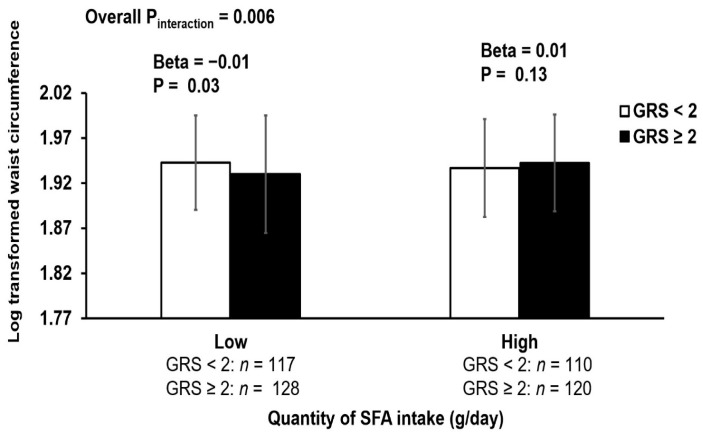
Interaction of GRS with SFA intake on log-transformed waist circumference. *p* values adjusted for age, sex, type 2 diabetes, duration of diabetes, anti-diabetic medication, smoking status, and alcohol intake. Low (≤23.2) and high (>23.2) refer to lower or equal to median and higher than median intake of SFA (g/day) respectively. In the low SFA intake group (≤23.2 g/day), individuals carrying 2 or more risk alleles had a smaller waist circumference compared to those carrying less than 2 risk alleles (Beta = −0.01, *p* = 0.03), and in the high SFA intake group (>23.2 g/day), there was no significant difference in waist circumference between participants carrying 2 or more risk alleles and those carrying less than 2 risk alleles.

A significant interaction was observed between GRS and total fat intake on WC (P_interaction_ = 0.03) after adjusting for age, sex, T2D, duration of diabetes, anti-diabetic medication, smoking status, alcohol intake, and total energy intake (Table 2). When individuals were stratified based on the median intake of total fat, there were no significant associations between GRS and total fat intake on WC, and when sub-types of fat were investigated (Figure 2), there was a significant interaction of GRS with SFA intake on WC (P_interaction_ = 0.006) and MUFA intake on WC (P_interaction_ = 0.004). In the low SFA intake group (≤23.2 g/day), individuals carrying ≥2 risk alleles had a smaller WC compared to those carrying <2 risk alleles (Beta = −0.01 cm, *p* = 0.03), while in the high SFA intake group (>23.2 g/day), there was no significant difference in WC between participants carrying ≥2 risk alleles and those carrying <2 risk alleles. For those individuals carrying ≥2 risk alleles, a high SFA intake (>23.2 g/day) was significantly associated with a larger WC than a low SFA intake (≤23.2 g/day) (Beta = 0.02 cm, *p* = 0.02). When individuals were grouped based on the median MUFA intake, there was no association between GRS and MUFA intake on WC. To examine whether the interactions of GRS with fat intake and SFA intake on WC were mediated by lipids, we included the four lipid subfractions (HDL, LDL, TG and total cholesterol) as confounding factors in addition to other confounding factors and found that the interaction was no longer significant for total fat intake (P_interaction_ = 0.08), but it remained significant for SFA intake (P_interaction_ = 0.02).

## 4. Discussion

Our study has shown that SFA intake may modify the effect of lipid-pathway genes on central obesity in Asian Indians. Our findings indicate that the combined effect of *LPL* and *CETP* SNPs (rs327, rs3200218 and rs4783961) on obesity traits may be altered by SFA intake, where consumption of high amounts of SFA may increase the combined genetic risk of central obesity posed by *LPL* and *CETP* SNPs while a low intake of SFA may help to reduce this risk. These findings are of public health importance considering the burden of central obesity in Asian Indians [2,51,52,53,54]. Our results suggest that Asian Indians with a higher genetic risk for central obesity are responsive to SFA intake and could benefit from dietary modifications to help prevent central obesity in Asian Indians.

An examination of the fatty acid profile of commonly consumed foods in India showed that milk and milk products were the main source of SFA and the median intake of SFA was 8.7% of total energy intake per day [55]. However, some of the commonly consumed food, such as potato chips, contained high amounts of palmitic acid, which could be attributed to the type of cooking oil used in their preparation [55]. The WHO’s dietary guidelines [56] state that SFA consumption should be less than 10% of total energy intake, and the National Dietary Guidelines Consensus Group [57] recommends that for Asian Indians who have higher LDL concentration (≥100 mg/dL), SFA intake should be <7% of total energy intake per day. Moreover, intake of SFA at 8.6% of total energy was found to be associated with increased risk of T2D in Indians [55]. In the present study, the median intake of SFA was 8.5% of total energy intake, which is within the WHO’s dietary guidelines [56], but as Indians are predisposed to dyslipidaemia, reducing SFA even further as recommended by the National Dietary Guidelines Consensus Group [57] might help to prevent central obesity in individuals with a high genetic risk.

Abnormalities in lipid metabolism have been linked to the development of obesity [16,58]. We used a nutrigenetic approach to see if dietary intake can modify this link by employing a GRS from the two lipid pathway genes, *CETP* and *LPL*, which have been shown to have the strongest effect on lipid concentrations [21,24,25,27,28,29,32,59]. To account for the effect of T2D on lipid levels, we adjusted for T2D status, anti-diabetic medication, and duration of T2D in our analysis. We found significant interactions between GRS and total fat, SFA and MUFA intake on WC, where a low intake of SFA (≤23.2 g/day) was found to be associated with a smaller WC in individuals with a higher genetic risk compared to those with a lower genetic risk. We also found that a high SFA intake (>23.2 g/day) was significantly associated with a larger WC than a low SFA intake (≤23.2 g/day) in individuals with a high genetic risk. Our findings are in agreement with the results of a double-blind, randomized, crossover, controlled-feeding trial performed in 101 participants from Canada and the United States [16] where consumption of a diet low in SFA and high in unsaturated fatty acids resulted in increased serum-mediated cholesterol efflux which showed a negative association with WC (Beta = −0.25, *p* = 0.01) and abdominal adiposity (Beta = −0.33, *p* = 0.02). A parallel controlled-feeding trial performed in 20 individuals who were centrally overweight [15] also showed that consumption of a high SFA diet resulted in an increase in the expression of inflammatory genes in adipose tissue and a decrease in the expression of genes involved in fatty acid β-oxidation and synthesis of triglycerides, which could explain the increase in WC with a high SFA intake observed in our study. *LPL* was chosen as one of the candidate genes for the present nutrigenetic study, given that significant associations between *LPL* SNPs and obesity traits have been reported by previous studies in addition to their association with lipid traits. In a case-control study of 944 Koreans [48], the *LPL* SNP rs3200218, which is in the 3′-UTR, was shown to be associated with WHR (*p* = 0.009), and in a previous study in CURES participants [33], carriers of the minor allele (G) of *LPL* SNP rs1800590 had a larger WC (*p* = 0.03) and higher BMI (*p* = 0.003) compared to those carrying two copies of the major allele (T). Increased risk of common obesity (2.73-fold increase) among carriers of the minor allele of *LPL* rs1800590 was also observed in Northern Indians [47]. Furthermore, LPL is a rate-restricting enzyme for the hydrolysis of TG in chylomicrons and VLDL [11], and it has been suggested that the level of LPL activity in muscle relative to that in adipose tissue determines body mass composition and contributes to obesity by influencing the rate at which fatty acids derived from TG are used or stored [10]. This suggests that SNPs that alter LPL activity in muscle and adipose tissue could affect obesity related traits. It has also been shown that SFAs are associated with a lower postprandial oxidation rate [60] and decreased energy expenditure [61] than MUFA.

Another important candidate gene for the study is *CETP*, the SNPs in which have been reported to influence obesity and lipid-related traits. The ‘A’ allele of the SNP rs4783961 has been shown to influence the concentration of CETP mass in plasma by producing binding motifs for transcription factor SP3, which modulates *CETP* promoter activity [62,63], but studies examining the association of rs4783961 with obesity traits are limited. However, the ‘A’ allele of rs4783961 has been linked to higher HDL concentration in Taiwanese [64] (an increase of 1.71 mg/dL per allele, standard error (SE) = 0.52; *p* = 0.001) and African Americans [22] (Beta = 4.6, SE = 1.3; *p* = 0.0009). A study involving 10,366 African American, 26,647 European American, 1410 Hispanics and 717 Chinese American participants from nine cohorts [65] also reported that the ‘A’ allele of rs4783961 was associated with increased HDL concentration in all the cohorts, but the effect size was larger in African Americans (0.17 to 0.24) than in European Americans (0.09 to 0.15) (*p* = 2 × 10^−10^). The mechanism under which rs4783961 affects obesity traits are unclear, although it has been proposed that *CETP* SNPs might affect deposition of fat in visceral adipose tissue by being in linkage with SNPs of other genes [65]. Nonetheless, association of other *CETP* SNPs with obesity traits have been previously reported. A cross-sectional study of 1005 Spanish individuals who were obese [66] reported that participants carrying the ‘A’ allele of *CETP* SNP rs1800777 compared to non-carriers had higher WC (Delta: 5.6 ± 2.1 cm; *p* = 0.02), WHR (Delta: 0.04 ± 0.01 cm; *p* = 0.01) and fat mass (Delta: 4.4 ± 1.1 kg; *p* = 0.04). Similarly, a study performed in 571 Chinese individuals [34] observed that participants with the ‘GT’ genotype of *CETP* SNP rs3764261 had a reduced risk of central obesity (Odds ratio (OR) = 0.631, 95% confidence interval (CI) = 0.460–0.865; *p* = 0.004), and a study involving 3575 Dutch participants [67] reported that the minor allele of *CETP* SNP rs5882 was associated with a decreased prevalence of central obesity (OR = 0.90, 95% CI = 0.83–0.97; *p* = 0.007).

Our findings of significant interactions between GRS and dietary fat intake on WC are consistent with a previous study [58]. This study [58], which consisted of 199 overweight/obese Spanish adolescents and involved a weight loss intervention, showed that each minor allele of *CETP* SNP rs1800777 was associated with a −1.4 kg decrease in body weight after 10 weeks (*p* = 1.5 × 10^−4^). Studies examining CETP and obesity have mainly focused on the impact of body weight on CETP mass and activity [68,69,70]. A study involving 21 morbidly obese female participants (BMI > 40 kg/m^2^) [68] who underwent a weight loss procedure concluded that weight loss was associated with a marked decrease in CETP mass and activity. Another study involving 51 normal weight individuals [70] also reported that participants with a body weight of around 46 kg had 15% lower serum CETP compared to those with a body weight of about 55 kg. However, an anti-adipogenic effect of CETP in the presence of apolipoprotein CIII (apoCIII) was reported by an animal study involving *CETP* and *apoCIII* transgenic mice [71], where obesity induced by a high-fat diet was reversed by the expression of *CETP*. As this study did not look at *CETP* SNPs, it is unclear whether different *CETP* SNPs will have the same effect. Individually, the SNPs in our study did not show any significant interaction with dietary factors. The discrepancies in findings between our study and others could be because of allele frequencies and effect sizes which differ between populations [1,32]. Another plausible explanation is differences in dietary pattern and the methods used to assess dietary intake [1]. Moreover, a systematic review of observational studies [72] concluded that SFAs were not linked to CVDs, and an analysis of data from randomized controlled trials [73] indicated that replacing SFA with linoleic acid was effective in lowering total cholesterol but there was no benefit in terms of lower risk of CVDs or death. However, large cohort studies [74,75] have indicated that the effect of SFA is dependent on the type and food sources of SFA. The European Prospective Investigation into Cancer and Nutrition—Netherlands (EPIC—NL) cohort study of 37,421 participants [74] observed that total dietary SFA had no association with T2D, but SFA derived from cheese and long-chain SFAs were negatively associated with T2D. The EPIC-InterAct case-cohort study of 27,296 participants [75] also reported that even-chain SFAs including palmitic acid and myristic acid had a positive association with T2D, while odd-chain and longer-chain SFAs had a negative association with T2D.

The strength of our study is the use of a GRS based on two established lipid pathway genes in a well characterised population. Our study is the first of its kind to investigate the link between lipids and obesity from a nutrigenetic perspective. Another strength is the use of validated questionnaires and the robust sensitivity analysis incorporating conventional risk factors including alcohol consumption and smoking as confounding factors. Nonetheless, our study has several limitations. The small sample size could have influenced the lack of association between GRS and the measured outcomes (lipids and obesity). Another limitation is that we did not investigate different types or sources of SFAs. As this is a cross-sectional study, it is not possible to determine causality between fat intake and WC. Despite our robust sensitivity analysis, we cannot rule out residual confounding from unidentified factors [1]. However, we were able to replicate previously reported interactions between GRS and fat intake on WC.

## 5. Conclusions

Our findings suggest that dietary fatty acid intake may modify the effect of SNPs in lipid-pathway genes on central obesity in Asian Indians. The results indicate that a diet low in SFA might help to reduce the genetic risk of central obesity while a high SFA diet might increase the genetic risk of central obesity in Asian Indians. These findings support the WHO’s dietary guidelines for preventing unhealthy weight gain by limiting SFA intake to less than 10% of total energy intake, and they indicate that personalised nutrition based on GRS might be an effective strategy for the management of central obesity in Asian Indians who have a high genetic risk, but additional studies with large sample sizes are needed to confirm our findings.

## Figures and Tables

**Table 1:** Characteristics of the study participants.

	All Participants (*n* = 497)	GRS < 2 (*n* = 239)	GRS ≥ 2 (*n* = 258)	*p* Value *
Age (years)	44 ± 10	45 ± 10	44 ± 9	0.34
Sex [Men (%), Women (%)]	225 (45), 272 (55)	106 (47), 133 (49)	119 (53), 139 (51)	0.69
BMI (kg/m^2^)	24.6 ± 4.5	24.7 ± 4.7	24.4 ± 4.3	0.41
WC (cm)	87 ± 11	88 ± 12	87 ± 11	0.39
WHR	0.92 ± 0.08	0.92 ± 0.09	0.91 ± 0.08	0.57
Obese cases (%)	209 (42)	109 (52)	100 (48)	0.12
HDL (mg/dL)	42 ± 10	42 ± 10	42 ± 10	0.79
LDL (mg/dL)	119 ± 32	118 ± 32	119 ± 32	0.81
TG (mg/dL)	165 ± 150	166 ± 120	164 ± 173	0.87
Total cholesterol (mg/dL)	191 ± 40	192 ± 42	190 ± 38	0.64
Systolic BP (mmHg)	122 ± 20	123 ± 22	120 ± 18	0.15
Diastolic BP (mmHg)	76 ± 11	76 ± 12	75 ± 11	0.60
Fasting plasma glucose (mg/dL)	126 ± 65	126 ± 64	127 ± 67	0.79
Fasting serum insulin (μIU/mL)	9 ± 6	9 ± 6	9 ± 7	0.89
Insulin resistance	3 ± 2	3 ± 2	2 ± 2	0.44
HbA1c (%)	7 ± 2	7 ± 2	7 ± 2	0.91
Fat (g)	67 ± 27	67 ± 26	67 ± 27	0.83
Carbohydrate (g)	410 ± 136	410 ± 134	411 ± 138	0.92
Protein (g)	72 ± 24	73 ± 24	72 ± 23	0.63
Dietary fibre (g)	32 ± 12	32 ± 12	32 ± 11	0.77
Energy (kcal/day)	2560 ± 822	2560 ± 809	2559 ± 834	0.99
Total SFA (g)	25 ± 11	25 ± 11	25 ± 11	0.91
Total MUFA (g)	20 ± 8	20 ± 8	21 ± 9	0.79
Total PUFA (g)	19 ± 9	18 ± 9	19 ± 10	0.77
Plant protein (g/day)	41 ± 14	40 ± 13	42 ± 14	0.23
Animal protein (g/day)	23 ± 13	23 ± 12	22 ± 13	0.75
Smokers (%)	88 (18)	33 (38)	55 (63)	0.03
Alcohol drinkers (%)	123 (25)	52 (42)	71 (58)	0.14
T2D cases (%)	260 (52)	131 (50.4)	129 (49.6)	0.28

Data are mean ± standard deviation or frequencies where appropriate. * *p* values for the differences in means/frequencies between participants with low GRS and those with high GRS. *p* values were calculated using independent sample t test for continuous variables and Chi-square test for categorical variables. BMI—body mass index; WC—waist circumference; WHR—waist hip ratio; HDL—high-density lipoprotein cholesterol; LDL—low-density lipoprotein cholesterol; TG—triglycerides; HbA1c—glycated haemoglobin; SFA—saturated fatty acids; MUFA—monounsaturated fatty acids; PUFA—polyunsaturated fatty acids.

**Table 2:** Interaction of GRS with dietary factors on blood lipids, blood pressure, obesity-related traits, and obesity.

Trait	GRS ∗ Fat (g)	GRS ∗ Carbohydrate (g)	GRS ∗ Protein (g)	GRS ∗ Dietary Fibre (g)
	Beta Coefficient ± SE (P_interaction_)	Beta Coefficient ± SE (P_interaction_)	Beta Coefficient ± SE (P_interaction_)	Beta Coefficient ± SE (P_interaction_)
**BMI (kg/m^2^)**	0.05 ± 0.04 (0.21) ^a^	0.04 ± 0.05 (0.36) ^a^	0.04 ± 0.05 (0.35) ^a^	−0.01 ± 0.04 (0.77) ^a^
**WC (cm)**	0.06 ± 0.03 (0.03) ^a^	0.05 ± 0.03 (0.18) ^a^	0.07 ± 0.04 (0.07) ^a^	0.00 ± 0.03 (0.93) ^a^
**Waist hip ratio**	0.01 ± 0.02 (0.52) ^b^	0.00 ± 0.02 (0.98) ^b^	0.01 ± 0.02 (0.58) ^b^	−0.01 ± 0.02 (0.62) ^b^
**Common obesity**	−1.76 ± 1.14 (0.12) ^a^	0.10 ± 0.08 (0.20) ^a^	−2.52 ± 1.41 (0.08) ^a^	−0.35 ± 1.26 (0.78) ^a^
**HDL (mg/dL)**	−0.04 ± 0.05 (0.42) ^b^	−0.07 ± 0.06 (0.23) ^b^	−0.07 ± 0.06 (0.21) ^b^	−0.04 ± 0.05 (0.47) ^b^
**LDL (mg/dL)**	0.02 ± 0.06 (0.82) ^b^	0.02 ± 0.08 (0.79) ^b^	−0.01 ± 0.08 (0.90) ^b^	−0.02 ± 0.07 (0.81) ^b^
**TG (mg/dL)**	0.10 ± 0.12 (0.39) ^b^	−0.01 ± 0.15 (0.97) ^b^	−0.02 ± 0.15 (0.89) ^b^	0.08 ± 0.13 (0.57) ^b^
**Total cholesterol (mg/dL)**	0.02 ± 0.04 (0.70) ^b^	−0.00 ± 0.06 (0.98) ^b^	−0.02 ± 0.06 (0.65) ^b^	−0.00 ± 0.05 (0.98) ^b^
**Systolic BP (mmHg)**	0.03 ± 0.03 (0.35) ^b^	0.03 ± 0.04 (0.49) ^b^	0.03 ± 0.04 (0.48) ^b^	0.04 ± 0.03 (0.25) ^b^
**Diastolic BP (mmHg)**	0.02 ± 0.03 (0.50) ^b^	0.01 ± 0.04 (0.87) ^b^	0.03 ± 0.04 (0.51) ^b^	0.01 ± 0.04 (0.72) ^b^

GRS—genetic risk score; BMI—body mass index; WC—waist circumference; HDL—high-density lipoprotein cholesterol; LDL—low-density lipoprotein cholesterol; TG—triglycerides. *p* values were obtained from linear regression analysis for continuous traits and logistic regression analysis for obesity. ^a^ *p* values adjusted for age, sex, type 2 diabetes, duration of diabetes, anti-diabetic medication, smoking status, alcohol intake, and total energy intake. ^b^ *p* values adjusted for age, sex, BMI, type 2 diabetes, duration of diabetes, anti-diabetic medication, smoking status, alcohol intake, and total energy intake. Log-transformed variables were used for the analysis. *p*-value in bold represents statistically significant interaction.

## Data Availability

The data presented in this study are available on request from the corresponding author. The data are not publicly available due to [ethical reasons].

## Supplementary Materials

The following supporting information can be downloaded at: https://www.mdpi.com/article/10.3390/nu14132713/s1, Table S1: Allele Frequencies and Hardy–Weinberg Equilibrium *p* value, Table S2: Association of GRS with blood lipids, blood pressure and obesity-related traits, Table S3: Association of GRS with obesity.
