# Supplementary material for: Impact of Lipid Genetic Risk Score and Saturated Fatty Acid Intake on Central Obesity in an Asian Indian Population
Source: Nutrients. 2022 Jun 29;14(13):2713. doi: 10.3390/nu14132713 (PMC9269337; doi:10.3390/nu14132713)
Supplement: Supplementary file 1 [file nutrients-14-02713-s001.zip › nutrients-1759233-supplementary.pdf]

Supplementary Table S1 Allele Frequencies and Hardy-Weinberg Equilibrium *P*-value (N = 497)

Gene & SNP	Genotype Count	Allele Frequencies (%)	Hardy-Weinberg Equilibrium *P*-value
CETP rs4783961	GG = 152<br>GA = 241<br>AA = 104	G = 55<br>A = 45	0.64
LPL rs327	TT = 286<br>TG = 190<br>GG = 21	T = 77<br>G = 23	0.13
LPL rs3200218	AA = 407<br>AG = 83<br>GG = 7	A = 90<br>G = 10	0.25
LPL T93G (rs1800590)	TT = 480<br>TG = 17<br>GG = 0	T = 98<br>**G = 2**	0.70
LPL rs268	GG = 472<br>GA = 25<br>AA = 0	G = 97<br>**A = 3**	0.57

Values in bold have a minor allele frequency less than 5%. SNP – single nucleotide polymorphism; *CETP* – Cholesteryl ester transfer protein; *LPL* – lipoprotein lipase.

Supplementary Table S2 Association of GRS with blood lipids, blood pressure and obesity-related traits

**Trait**	**Mean ± Standard Deviation**		***P* value**
	**GRS < 2 (N = 239)**	**GRS ≥ 2 (N = 258)**	
**BMI (Kg/m^2)**	24.3 ± 1.2	24.0 ± 1.2	0.70[a]
**Waist circumference (cm)**	87.1 ± 1.1	86.3 ± 1.1	0.65[a]
**Waist hip ratio**	0.90 ± 1.10	0.90 ± 1.10	0.73[b]
**Systolic BP (mmHg)**	121.3 ± 1.2	119.3 ± 1.2	0.51[b]
**Diastolic BP (mmHg)**	74.8 ± 1.2	74.4 ± 1.2	0.93[b]
**HDL (mg/dl)**	41.1 ± 1.3	41.4 ± 1.3	0.92[b]
**LDL (mg/dl)**	114.1 ± 1.3	114.7 ± 1.3	0.81[b]
**TG (mg/dl)**	138.4 ± 1.8	130.9 ± 1.8	0.43[b]
**Total cholesterol (mg/dl)**	188.2 ± 1.2	186.7 ± 1.2	0.74[b]

GRS – genetic risk score; BMI – body mass index; HDL – high-density lipoprotein cholesterol; LDL – low-density lipoprotein cholesterol; TG –triglycerides. *P* values were obtained from linear regression analysis using log-transformed variables.

[a] *P* values adjusted for age, sex, type 2 diabetes, duration of diabetes, anti-diabetic medication, smoking status, and alcohol intake.

[b] *P* values adjusted for age, sex, BMI, type 2 diabetes, duration of diabetes, anti-diabetic medication, smoking status, and alcohol intake. Log-transformed variables were used for the analysis.

Supplementary Table S3 Association of GRS with obesity

Trait	Odds ratio (95% C.I)	N		*P* value
		GRS < 2	GRS ≥ 2	
**Common obesity**	1.34 (0.89 – 2.01)	214	236	0.16
**Central obesity**	1.20 (0.81 – 1.80)	227	248	0.37

GRS – genetic risk score.
P values were obtained from logistic regression analysis, adjusted for age, sex, type 2 diabetes, duration of diabetes, anti-diabetic medication, smoking status, and alcohol intake. Log-transformed variables were used for the analysis.
